# Valsartan‐associated bullous pemphigoid initially presenting as erythema multiforme

**DOI:** 10.1002/hsr2.452

**Published:** 2021-12-21

**Authors:** Zhe Gao, Yongping Cao, Xiaofang Zeng, Xiuzu Song

**Affiliations:** ^1^ Department of Dermatology The Third People's Hospital of Hangzhou Hangzhou China

## 1 INTRODUCTION

Bullous pemphigoid (BP), as an acquired autoimmune disease, is characterized by tense blisters on the erythematous or normal‐looking skin. More than 20% of the cases present as nonbullous phases.^1^ Extensive atypical BP presentations have been occasionally reported.^1^ Here, we report a case of valsartan‐associated erythema multiforme‐like BP (EMBP).

## 2 REPORT

**FIGURE 1:**
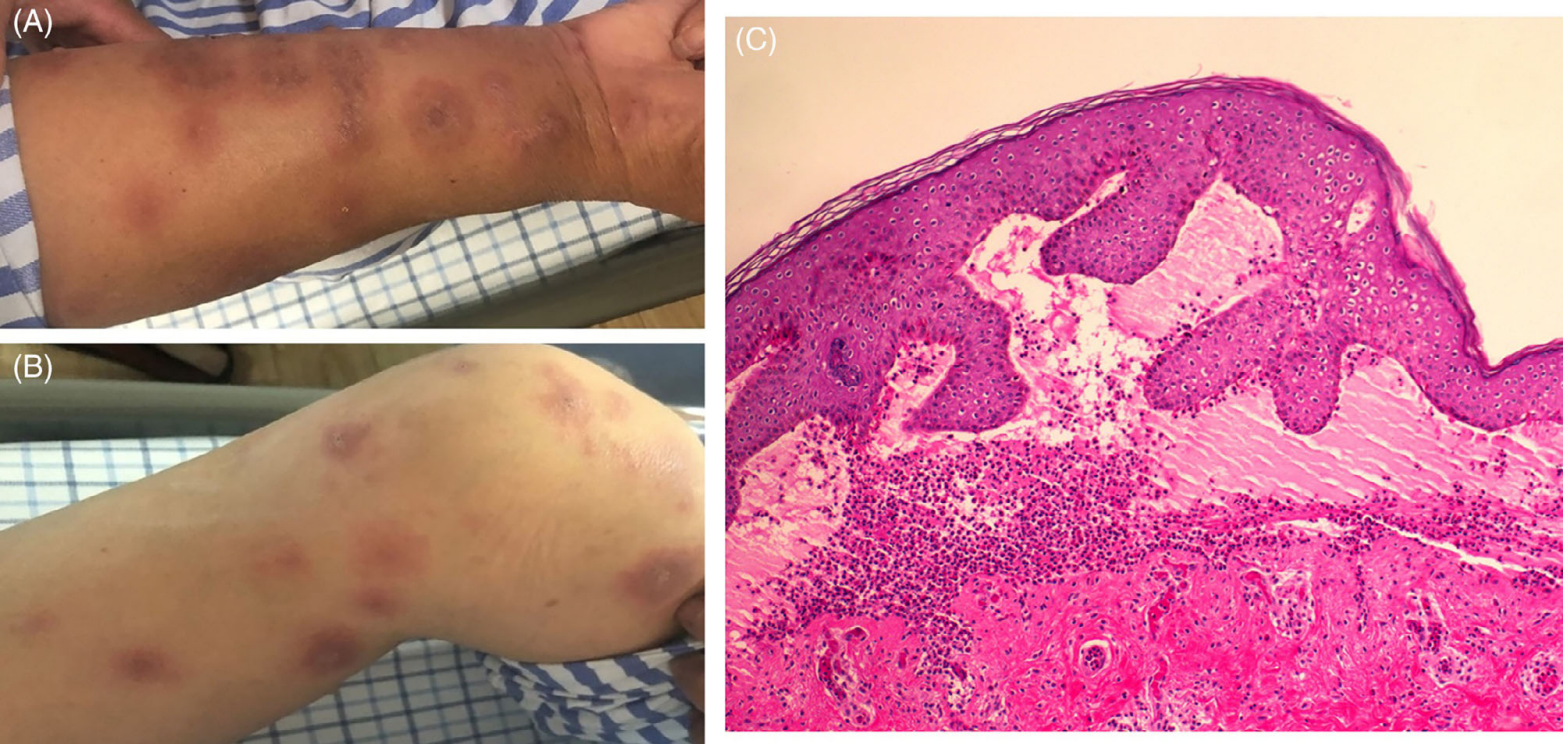
(A, B) Clinical presentation of targetoid erythematous lesions on limbs. (C) Central portion of the lesion showing subepidermal blister and dermal infiltrate with eosinophils and lymphocytes (HE × 100)

A 62‐year‐old woman with itchy targetoid erythematous lesions on her hand, arms, and thighs for 20 days was enrolled (Figure 1A,B). She had a history of hypertension and hyperlipidemia and more than 3‐year medication history of nifedipine and simvastatin, with no history of other diseases and drugs. One month before her presentation, the nifedipine regimen was changed to valsartan. The histology showed subepidermal splitting and eosinophil infiltration in the dermis (Figure 1C). The direct immunofluorescence observed a linear deposition of IgG and C3 along the basement membrane zone.

The diagnosis of EMBP was established. Valsartan suspected of causing the issue had, hence, was discontinued. She was treated with systemic prednisolone (0.75 mg/kg/day) and topical halometasone cream (25 g/day). Moreover, methotrexate (12.5 mg/week) was added on the fifth day due to the progression of new erythema. One week after methotrexate addition, the condition was controlled.

## 3 DISCUSSION

Only 10 cases of EMBP have been reported in the English literature.^2^, ^3^ Most patients (including our case) had no mucosal involvement, and it was controlled within 1 month after cessation of predisposing factors.

Valsartan's, as an angiotensin II receptor blocker (ARB), common side effects are malaise/lassitude, dizziness, cough, and abdominal pain. Moreover, some adverse skin reactions (such as urticaria, linear lichenoid, and mucosal BP) have also been occasionally reported.^4^, ^5^ However, this is the first report focusing on valsartan or even ARB inducing EMBP.

## 4 CONCLUSION

Drug‐induced diagnoses should be always considered in BP, especially when the lesions are atypical.

## FUNDING

This study was supported by the National Natural Science Foundation of China (81872517) and Basic Public Welfare Research Project of Zhejiang Province (LGF18H110001).

## CONFLICT OF INTEREST

The authors declare no conflicts of interest.

## AUTHOR CONTRIBUTIONS

Conceptualization: Zhe Gao and Xiuzu Song.

Resources: Yongping Cao and Xiaofang Zeng.

Supervision: Xiuzu Song.

Writing—Original Draft Preparation: Zhe Gao and Yongping Cao.

Writing—Review & Editing: Zhe Gao and Xiaofang Zeng.

All authors have read and approved the final version of the manuscript.

All authors had full access to all of the data in this study and take complete responsibility for the integrity of the data and the accuracy of the data analysis.

## TRANSPARENCY STATEMENT

The authors affirm that this manuscript is an honest, accurate, and transparent account of the study being reported; that no important aspects of the study have been omitted; and that any discrepancies from the study as planned (and, if relevant, registered) have been explained.

## ETHICS STATEMENT

Written informed consent was obtained from the participant, and the study was conducted in accordance with the principles of the Declaration of Helsinki (2013).

## Data Availability

The authors confirm that the data supporting the findings of this study are available within the article.
